# Ozonated Sunflower Oil Exerted Protective Effect for Embryo and Cell Survival via Potent Reduction Power and Antioxidant Activity in HDL with Strong Antimicrobial Activity

**DOI:** 10.3390/antiox10111651

**Published:** 2021-10-21

**Authors:** Kyung-Hyun Cho, Dae-Jin Kang, Hyo-Seon Nam, Ju-Hyun Kim, Su-Young Kim, Jung-Ok Lee, Beom-Joon Kim

**Affiliations:** 1 Raydel Research Institute, Medical Innovation Complex, Daegu 41061, Korea; daejin@rainbownature.com (D.-J.K.); sun91120@rainbownature.com (H.-S.N.); aksk1694@rainbownature.com (J.-H.K.); 2 LipoLab, Yeungnam University, Gyeongsan 712-749, Korea; 3 Department of Dermatology, College of Medicine, Chung-Ang University, Seoul 06974, Korea; sykim940714@naver.com (S.-Y.K.); misocell@gmail.com (J.-O.L.); BeomJoon74@gmail.com (B.-J.K.)

**Keywords:** ozonated sunflower oil (OSO), high-density lipoproteins (HDL), antioxidant, low-density lipoproteins, zebrafish embryo

## Abstract

Ozonated sunflower oil (OSO) has potent antimicrobial effects, making it useful for topical applications to treat various skin diseases. On the other hand, regarding mechanistic insight, the antioxidant activity and cytoprotective effects of OSO are relatively less known. The current study compared the antioxidant ability and protective ability of OSO on cells and embryos against oxidative stress, such as H_2_O_2_ and oxidized low-density lipoproteins (oxLDL), to investigate its potential applications for wound-healing and anti-infection. OSO showed potent radical scavenging activity and ferric ion reduction ability that was up to 35% and 42% stronger than sunflower oil (SO) as a control in a dose-dependent manner. Measurement of the wavelength-maximum fluorescence (WMF) of high-density lipoproteins (HDL) revealed different behavior between OSO and SO treatment (final 1–16%). The OSO treatment caused a 12 nm red shift of Trp movement from 345 nm (at 0%) to 357 nm (at 16%), while SO caused a 12 nm blue shift of Trp movement from 345 nm (at 0%) to 333 nm (at 16%). The fluorescence intensity of HDL_3_ was diminished remarkably by the OSO treatment by up to 80% from the initial level, while SO-treated HDL did not. OSO-treated HDL_3_ showed slower electromobility with stronger band intensity and bigger HDL particle sizes than those of SO-treated HDL_3_. The paraoxonase-1 (PON-1) activity of HDL_3_ was enhanced by a co-treatment of OSO that was up to 2.3 times higher than HDL_3_ alone in a dose-dependent manner, whereas the co-treatment of SO even inhibited the PON activity. The cell viability of RAW264.7 by the OSO treatment was 3.3 times higher than the SO treatment at a high dose range (from 10% to 50%, final). The OSO also exhibited more cytoprotective effects than SO in brain microglial cells in the presence of H_2_O_2_ (final 0.03%); treatment with OSO impeded apoptosis and reduced ROS production more than an SO treatment did. In the presence of H_2_O_2_ alone, 86 ± 5% of the embryos were killed by cell explosion after 24 h, but a co-treatment of OSO (final 4%) resulted in almost no embryo death (98% survivability). Injection of oxLDL (15 ng of protein) into zebrafish embryos caused acute death, while the co-injection of OSO (final 2%) resulted in 2.8 times higher survivability than oxLDL alone. These results suggest new effects of ozonated oil, such as enhanced antioxidant activity, more cytoprotective ability, and higher embryo protection against oxidative stress. These results may be useful in developing new methods for the quality control of ozonated oil and an assessment of its efficacy.

## 1. Introduction

Ozone, a highly unstable gas, has been used for medical treatment owing to its strong oxidizing capacity. Ozone can initiate the oxidation of the plasma membrane of microorganisms from viruses to fungi and eventually destroys these microorganisms [1]. Therefore, many earlier ozone therapies mainly focused on disinfection and sanitization because of the potent germicidal activities of ozone [2]. Ozone has recently been used in dermatology clinics to treat allergic disease, wound healing, and ulcer recovery [3]. Interestingly, many ozone therapies have improved the blood lipid profiles in animals and humans. Male rats exposed to aerosol ozone (0, 1, 1.75, 3.0 ppm) for several days in room air caused an increase in HDL-C and a decrease in LDL-C [4]. In psoriatic patients, ozonated autohemotherapy increased the serum HDL-C level and decreased the total cholesterol, LDL-C level, and triglyceride (TG) level with the suppression of inflammatory markers, such as tumor necrosis factor (TNF) and interleukin (IL) [5].

Various ozonated products, including those based on water and edible oils, have been mainly developed based on vegetable oils, olive oil, and sunflower oil and have been applied human and veterinary medicine and cosmetics [6]. Although the mechanism is still not fully understood, ozonated water promotes the regeneration of zebrafish caudal fin by regulating inflammatory responses, such as the reduction of TNF-α and IL-10 [7]. Ozonated oils are also used in many diseases, such as joint and skin disorders. Ozonized sunflower oil (OSO) exerts antibacterial and antifungal activities with germicidal properties [8,9]. The ozonolysis of supercoiled DNA, proteolysis, and double bond cleavage at the unsaturated fatty acids have been suggested as putative mechanisms of wide-range disinfection [10]. OSO, oleozon^®^, displayed high efficacy in treating onychomycosis [11] and tinea pedis [12]. Recently, the tissue regeneration effect of OSO by preventing hypergranulation tissue and infection was also reported in an equine model [13].

Despite the many beneficial effects of OSO, it is unclear why OSO exerted favorable physiological effects on tissue regeneration and wound healing [14]. Tissue regeneration activity should be accompanied by sufficient antioxidant activity, such as the removal of radical superoxide and electron donation. Thus far, most reports and patents have focused on the antimicrobial and antifungal activities of ozonated oil for topical applications and disinfection [15]. Consequently, few studies have examined the mechanism for the antioxidant activity of OSO, which is linked with its cell protection and tissue regeneration abilities.

Furthermore, because the physiological roles of OSO in the lipid and protein metabolism are unclear, it is difficult to explain the wound-healing effect of OSO because cholesterol is essential for tissue regeneration. The potential antioxidant activity of ozonated oil has not been considered because many studies have only focused on its antibacterial and antifungal activities. More importantly, there has been no study of ozonated oil quality regarding its basic functionality. The current method of comparing the antibacterial activity of ozonated oil is time-consuming and laborious work. Therefore, a rapid and straightforward method is needed to evaluate the quality status and to maintain the efficacy of ozonated oil from various brands.

This study examined the basic mechanism of OSO regarding antioxidant activity, lipoprotein stability, cytoprotective properties, and embryo protection from oxidative stress as well as germicidal activities. The protective effects against infectious and inflammatory diseases were examined to provide more functional insight into ozonated oil.

## 2. Materials and Methods

### 2.1. Materials

Ozonated sunflower seed oil (Raydel^®^ Bodyone Flambo oil) was provided by Rainbow and Nature Pty, Ltd. (Thornleigh, NSW, Australia). The physicochemical characteristics of the OSO (Raydel^®^ Bodyone Flambo oil) revealed the typical range of Oleozon^®^, as described elsewhere [11]: 783.4 mmol of peroxide/kg (range 500–800 mmol/kg) and viscosity of 131.5 mPa.s (90–350 mPa.s) with optimal acidity of 2.42 mg KOH/g. Commercially available sunflower oil (SO) was purchased from a local market (Ondoliva oil, Urzante, Spain). The pH levels of the OSO and SO were measured with a calibrated pH meter (Thermo 920A pH/ISE meter, Waltham, MA, USA) and were 3.0 and 4.8 at room temperature, around 25 °C, respectively, indicating that OSO was approximately 63 times more acidic than SO. Under acidic conditions (pH 3.0–4.0), ozone is stable, and its decomposition rate is relatively slow, as previously reported [16].

### 2.2. Radical Scavenging Assay

A solution of diphenyl-1-picrylhydrazyl (DPPH) free radicals was prepared by dissolving 2.4 mg of DPPH in 100 mL methanol using the standard method [17]. The DPPH solution (0.95 mL) in ethanol was then mixed with OSO or SO (final 10% and 20%) as a source of antioxidants. The mixture was continuously observed at 517 nm for 60 min at 25 °C using a UV-2600i spectrophotometer (Shimadzu, Kyoto, Japan) with Labsolutions software UV-Vis 1.11 (Shimadzu, Kyoto, Japan).

### 2.3. Ferric Ion Reducing Ability Assay

The ferric ion reducing ability (FRA) was determined using the method reported by Benzie and Strain [18]. Briefly, the FRA reagents were freshly prepared by mixing 20 mL of 0.2 M acetate buffer (pH 3.6), 2.5 mL of 10 mM 2,4,6-tripyridyl-S-triazine (Fluka Chemicals, Buchs, Switzerland) and 2.5 mL of 20 mM FeCl_3_∙6H_2_O. The antioxidant activities of OSO (final 1, 2, and 4%) were estimated by measuring the increase in absorbance induced by the ferrous ions that were generated. Freshly prepared FRA reagent (300 μL) was mixed with OSO and SO as an antioxidant source. The FRA was then determined by measuring the absorbance at 593 nm every two min during the 60 min period at 25 °C using a UV-2600i spectrophotometer.

### 2.4. Purification of Human Lipoproteins

LDL (1.019 < d < 1.063), HDL_2_ (1.063 < d < 1.125), and HDL_3_ (1.125 < d < 1.225) were isolated via sequential ultracentrifugation from the blood of a young human male (25-years-old), who voluntarily donated blood at the Blood Bank of Yeungnam University Medical Center after fasting overnight. The density was adjusted appropriately by adding NaCl and NaBr, as detailed previously [19], and the procedures were conducted using the standard protocols [20]. Samples were centrifuged for 24 h at 10 °C at 100,000× *g* using a Himac CP-100 NX (Hitachi, Tokyo, Japan) at the LipoLab, Yeungnam University (Gyeongsan, Korea).

### 2.5. Production of Oxidized LDL

Oxidized LDL (oxLDL) was produced by incubating the LDL fraction with CuSO_4_ (final concentration, 10 μM) for 4 h at 37 °C. The oxLDL was then filtered (0.2 μm) and analyzed using thiobarbituric acid-reacting substances (TBARS) assay to determine the extent of oxidation, as described previously [21].

### 2.6. Wavelength Maximum Fluorescence of HDL

The change in the secondary structure upon treatment with OSO was observed at the wavelengths of maximum fluorescence (WMF) of the tryptophan residues in HDL_3_. The WMF was determined from the uncorrected spectra obtained on an FL6500 spectrofluorometer (Perkin-Elmer, Norwalk, CT, USA) using Spectrum FL software version 1.2.0.583 (Perkin-Elmer) using a 1 cm path-length Suprasil quartz cuvette (Fisher Scientific, Pittsburgh, PA, USA). The samples were excited at 295 nm to avoid tyrosine fluorescence. The emission spectra were scanned from 305 to 400 nm at room temperature.

### 2.7. Electrophoresis of HDL_3_

The relative electrophoretic mobility depends on the intact charge and three-dimensional structure of HDL. Hence, agarose gel electrophoresis was conducted with OSO- or SO-treated HDL_3_ (final concentration of oil 0, 2, 4, 8, and 16%) in the non-denatured state, according to a previous report [22].

### 2.8. Electron Microscopy

Transmission electron microscopy (TEM, Hitachi, model HT-7800; Ibaraki, Japan) was performed at 80 kV. HDL_3_ was negatively stained with 1% sodium phosphotungstate (PTA; pH 7.4) with a final protein concentration of 0.3 mg/mL in TBS. An amount of 5 μL of the HDL suspension was blotted with filter paper and was replaced immediately with a 5 μL droplet of 1% PTA. After a few seconds, the stained HDL fraction was blotted onto a Formvar carbon-coated 300 mesh copper grid and was air-dried. The shape and size of the HDL were determined by TEM at a magnification of 40,000× *g*, according to a previous report [23].

### 2.9. Paraoxonase Assay

The paraoxonase-1 (PON-1) activity toward paraoxon was determined by evaluating the hydrolysis of paraoxon to *p*-nitrophenol and diethylphosphate catalyzed by the enzyme [24]. Equally diluted HDL_3_ (20 μL, 2 mg/mL) was added to 180 μL of paraoxon-ethyl (Sigma Cat. No. D-9286) containing asolution (90 mM Tris-HCl/3.6 mM NaCl/2 mM CaCl_2_ (pH 8.5)) with either OSO or SO (final 1, 2, 4%). The PON-1 activity was then determined by measuring the initial velocity of *p*-nitrophenol production at 37 °C, as determined by measuring the absorbance at 415 nm (microplate reader, Bio-Rad model 680; Bio-Rad, Hercules, CA, USA).

### 2.10. Cell Viability Assay

Mouse monocyte (RAW264.7, ATCCTIB-71) and brain microglial cells (BV-2) were maintained in Dulbecco’s modified Eagle’s medium (DMEM) supplemented with 10% fetal bovine serum (FBS), 100 U/mL penicillin, and 100 μg/mL streptomycin. The RAW264.7 and BV-2 were cultured in the DMEM media and were maintained at 70% confluence at 37 °C in a humidified incubator containing 5% CO_2_. The cells were treated with the designated concentrations of OSO in DMSO and were incubated for 24–48 h. After incubation, the extent of apoptosis was measured by acridine orange (Sigma Cat# A9231) staining and was visualized by fluorescence detection (Ex = 502 nm, Em = 525 nm), as previously described [25]. The production of the reactive oxygen species (ROS) by the cells was observed by dihydroethidium (DHE) staining (Ex = 588 nm, Em = 605 nm), as reported elsewhere [26].

### 2.11. Zebrafish Embryo

Zebrafish and embryos were maintained using the standard protocols [27]. The maintenance of the zebrafish and procedures using zebrafish were approved by the Committee of Animal Care and Use of Yeungnam University (Gyeongsan, Korea). The fish were maintained in a system cage at 28 °C under a 10:14 h light cycle with the consumption of normal tetrabit (TetrabitGmbh D49304, 47.5% crude protein, 6.5% crude fat, 2.0% crude fiber, 10.5% crude ash), containing vitamin A (29,770 IU/kg), vitamin D3 (1860 IU/kg), vitamin E (200 mg/kg), and vitamin C (137 mg/kg); Melle, Germany).

### 2.12. Microinjection of Zebrafish Embryos

Embryos at one day post-fertilization (dpf) were individually injected with a microinjection using a pneumatic picopump (PV830; World Precision Instruments, Sarasota, FL, USA) equipped with a magnetic manipulator (MM33; Kantec, Bensenville, IL, USA) and a pulled microcapillary pipette-using device (PC-10; Narishigen, Tokyo, Japan). Bias was minimized by performing the injections at the same position on the yolk. When necessary for co-injection, immediately before the injection, the oxLDL (15 ng of protein) was mixed with OSO or SO (final 2%) in 100 nL. After the injection, live embryos were observed under a stereomicroscope (Motic SMZ 168; Hong Kong) and were photographed using a Motic cam2300 CCD camera.

### 2.13. Imaging of Reactive Oxygen Species (ROS)

After injecting OSO or SO with HDL, the reactive oxygen species (ROS) levels in the embryos were imaged by dihydroethidium (DHE, cat # 37291; BioChemika) staining, as described previously [26]. The image was obtained by fluorescence observation (Ex = 588 nm and Em = 605 nm) using a Nikon Eclipse TE2000 microscope (Tokyo, Japan).

### 2.14. Antimicrobial Activity Test

The bacterial and fungal strains were purchased from American Type Culture Collection (ATCC, Rockville, MD, USA), as listed in Table 1. *S. aureus* and *E. coli* were cultured in Tryptic soy broth, and *C. albicans* was cultured in yeast malt broth. *C. acnes* were cultured in modified reinforced *Clostridium* medium (ATCC medium 2107) under anaerobic conditions at 37 °C for 72 h. The bacterial and fungal suspension at an optical density at 600 nm (OD_600_) was adjusted to 0.5 using a microplate reader (Spectra Max i3x; Molecular Devices, San Jose, CA, USA) before use.

The adjusted suspension (100 μL) of these strains was spread on an agar plate. The oil was diluted at each concentration in dimethyl sulfoxide (DMSO), which was used as a negative control. Six mm paper disks impregnated with different dilutions of ozonized sunflower oil (1/2, 1/10, 1/20, 1/33, 1/400, 1/1000-diluted) were positioned on an agar plate to evaluate the antibacterial and antifungal effects of OSO. The diameters of the growth inhibition zone were measured after incubation at 37 °C for 24–72 h (72 h for *C. acnes*). These strains were inoculated into each growth medium. OSO was added to make a final concentration of 0.1, 0.5, 3, and 5% in each growth medium. The strains were incubated at 37 °C with shaking (200 rpm) for 24 h (72 h for *C. acnes*). After incubation, 100 μL of each strain suspension was spread on an agar plate and was incubated at 37 °C for 24–72 h. *C. albicans* (1 × 10^8^ cells) were treated with OSO at 0.1%, 0.5%, 3%, and 5% (final). After 1 h incubation at 37 °C, the viability was measured via trypan blue staining using microscopy (Leica DMi8, Leica Microsystems, Wetzlar, CMSGmbH, Germany)

### 2.15. Statistical Analysis

The data of this study are expressed as the mean ± SD from at least three independent experiments with duplicate samples. For the zebrafish embryo study, multiple groups were compared using one-way analysis of variance (ANOVA) using a Scheffe test. Statistical analysis was performed using the SPSS software program (version 23.0; SPSS, Inc., Chicago, IL, USA). A *p* value < 0.05 was considered significant.

## 3. Results

### 3.1. In Vitro Antioxidant Activities of OSO

**Figure 1:**
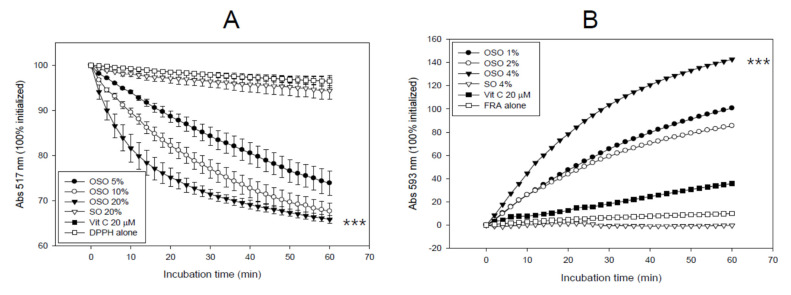
Antioxidant ability of ozonated sunflower oil (OSO). (**A**) DPPH radical scavenging activity. *** *p* < 0.005 compared to SO; (**B**) Ferric ion reduction ability. *** *p* < 0.005 compared to SO.

As shown in Figure 1A, the DPPH radical scavenging assay showed that OSO removed the radicals in a dose-dependent manner. The final 5, 10, and 20% treatments showed 27%, 33%, and 35% reduction of DPPH radicals, respectively, during 60 min of incubation, while SO (final 20%) only showed a 6% decrease in radical scavenging activity. Interestingly, the radical scavenging activity of OSO increased gradually and lasted for more than five days (data not shown), indicating that the antioxidant power of OSO could be maintained for long-term application owing to its strong hydrophobicity.

OSO (final 4%) showed the highest ferric ion reduction ability, with up to a 142% increase, while vitamin C (final 10 μM) showed a 35% increase, and SO showed no increase (Figure 1B). The FRA assay was conducted at low dosages (1, 2, and 4%) due to its insolubility in water, based on the FRA reagent. These results suggest that ozonated oil displayed potent antioxidant activity, including radical scavenging (Figure 1A) and ferric ion reduction ability (Figure 1B).

### 3.2. Movement of Trp Fluorescence in HDL

**Figure 2:**
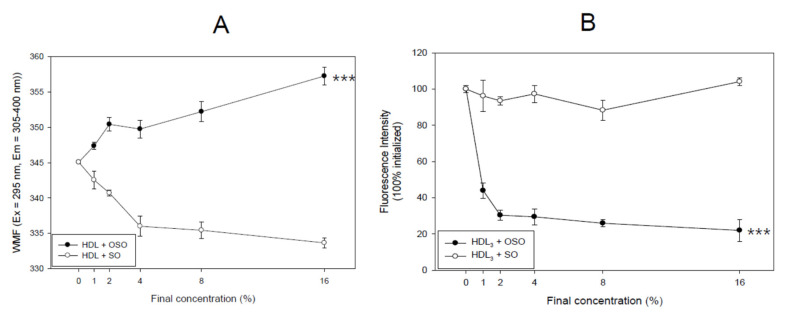
Measurement of the Trp fluorescence in HDL_3_. (**A**) Change in the wavelength-maximum fluorescence (WMF) depends on the ozonated sunflower oil (OSO) treatment. *** *p* < 0.005 between OSO and SO; (**B**) change in the fluorescence intensity (FI) dependent on OSO treatment. *** *p* < 0.005 between OSO and SO.

As shown in Figure 2A, Trp fluorescence scanning showed that OSO-treated HDL_3_ showed a 12 nm red-shift of the WMF around 357 nm (final 16% treated) than the control HDL_3_ around 345 nm. The OSO-treated HDL_3_ showed a gradual increase in the WMF from 347 nm (final 1%) to 357 nm (final 16%) in a dose-dependent manner. On the other hand, SO-treated HDL_3_ produced a 12 nm blue shift of the WMF to 333 nm (final 16% treated). The SO-treated HDL_3_ showed a gradual decrease in WMF from 342 nm (final 1%) to 333 nm (final 16% treated).

The fluorescence intensity (FI) of the WMF (Ex = 295, Em = 305–400) was decreased sharply by the OSO treatment (final 2%) in HDL_3_, an up to 70% decrease in FI from the initial level. The FI decreased gradually to an 80% reduction and was saturated at the 16% (final) treatment. By contrast, the SO treatment in HDL_3_ did not show a change in WMF that was similar to the initial level, as shown in Figure 2B. Compared to the initial level, 16% OSO treatment caused an almost 80% decrease in fluorescence, whereas the SO treatment in HDL_3_ did not. Overall, these results suggest that a putative oxygen species or singlet oxygen (^1^O_2_) in OSO caused the collisional quenching of the indole side chain of Trp fluorescence, the principal fluorophore of HDL and apoA-I.

### 3.3. Electrophoretic Mobility

**Figure 3:**
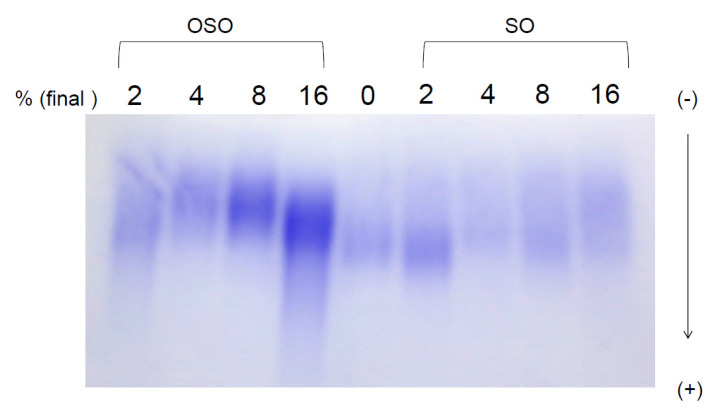
Electrophoretic profiles of HDL after incubation with OSO and SO (final 2, 6, 8, and 16%) for 72 h at 4 °C. The relative electrophoretic mobility was compared on 0.5% agarose gel, and the protein band was visualized by Coomassie blue staining.

The relative electrophoretic mobility of each HDL, which was incubated with OSO or SO (final 2, 4, 8, and 16%) for 72 h at 4 °C, was compared on 0.5% agarose gel in an undenatured state. As shown in Figure 3, OSO-treated HDL_3_ showed remarkably slower electromobility than HDL_3_ alone or SO-treated HDL_3_. Interestingly, OSO-treated HDL_3_ showed a much stronger protein band intensity in a dose-dependent manner than HDL_3_ alone, while all of the SO-treated HDL_3_ showed a much weaker and smeared band intensity. The final 16% OSO-treated HDL_3_ showed a 4.7- and 4.3-fold higher band intensity than that of the final 16% SO-treated HDL_3_ and HDL_3_ alone, respectively. On the agarose gel, the more oxidized LDL moved to the bottom of the gel more quickly and had more smear and weaker band intensity.

### 3.4. Particle Size of HDL

**Figure 4:**
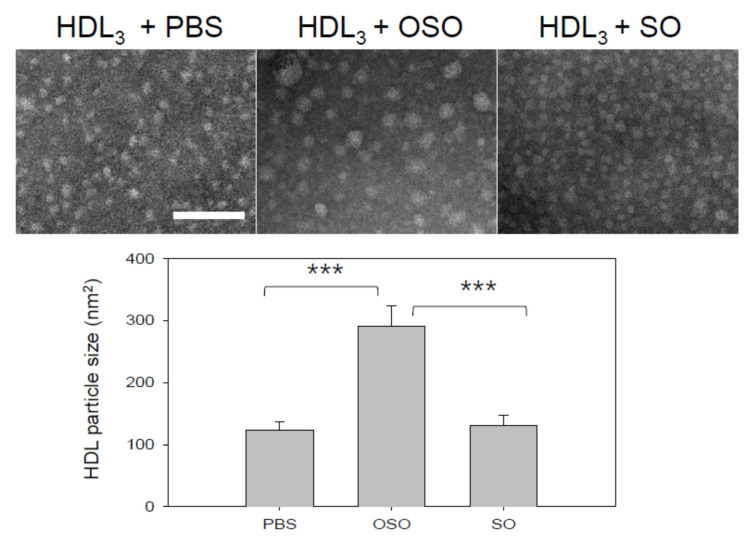
Transmission electron microscopic image of HDL_3_ under presence of OSO or SO (final 16%) with magnification of 40,000× *g*. OSO, ozonated sunflower oil; SO, sunflower oil; PBS, phosphate-buffered saline. *** *p* < 0.005.

Electron microscopic observation revealed that OSO-treated HDL_3_ (final 16% of oil) showed 2.3-fold and 2.2-fold bigger particle size than PBS-treated HDL_3_ and SO-treated HDL_3_, respectively, as shown in Figure 4. The mean size of the OSO-treated HDL_3_ was 291 ± 33 nm^2^, while the PBS-treated HDL_3_ and SO-treated HDL_3_ showed 124 ± 13 and 131 ± 16 nm^2^, respectively. HDL_3_ alone and SO-treated HDL_3_ showed similar particle diameters of around 12 ± 1 nm, while OSO-treated HDL_3_ showed 19 ± 2 nm diameters. These results indicate that OSO could induce structural changes in the HDL particle via s putative mechanism, which correlates well with the red-shift of WMF (Figure 2), the slower electromobility, and the stronger band intensity (Figure 3).

### 3.5. PON Activity of HDL_3_

**Figure 5:**
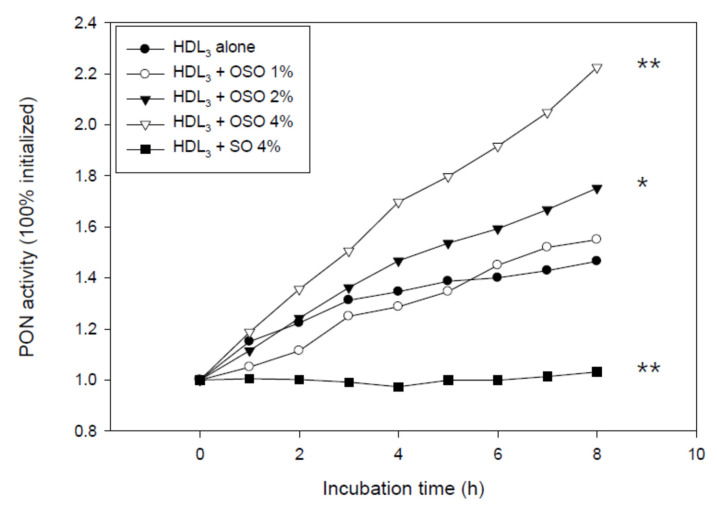
Paraoxonase (PON-1) activity of HDL_3_ in the presence of OSO and SO. OSO, ozonated sunflower oil; SO, sunflower oil; * *p* < 0.05 compared to HDL_3_ alone; ** *p* < 0.01 compared to HDL_3_ alone.

As shown in Figure 5, the OSO-treated HDL_3_ at final concentrations of 1%, 2%, and 4%, showed 6%, 19%, and 52% higher PON activity, respectively, than HDL_3_ alone during an 8 h incubation period, whereas SO-treated HDL_3_ lost the PON activity. This suggests that a putative component of OSO could enhance the PON activity in a dose-dependent manner. Similarly, the ozone-treated PON-1 activity was slightly increased at post 1 h and 20 h.

### 3.6. Cell Viability of Macrophage by OSO

**Figure 6:**
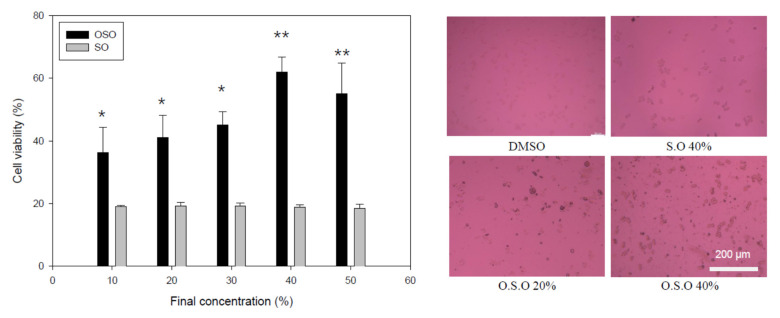
Cell viability and morphology of murine macrophage cells (Raw264.7) in the presence of ozonated sunflower oil. * *p* < 0.05 compared to SO; ** *p* < 0.01 compared with SO.

A high dose (final 10–50%) treatment of OSO into murine macrophage cells (Raw264.7) showed higher viability of around 36–61% cell survival than those of the SO treatment (~18–19% cell viability) (Figure 6A). Interestingly, a treatment of 10% to 40% OSO produced a gradual increase in viability in a dose-dependent manner. The highest viability was 61.9% survival with a healthy cell morphology (Figure 6B) at the 40% OSO treatment, suggesting that the high dose of OSO did not show cytotoxicity to mammalian macrophage cells.

### 3.7. Cytoprotective Activity of OSO against Oxidative Stress in BV-2 Cell

**Figure 7:**
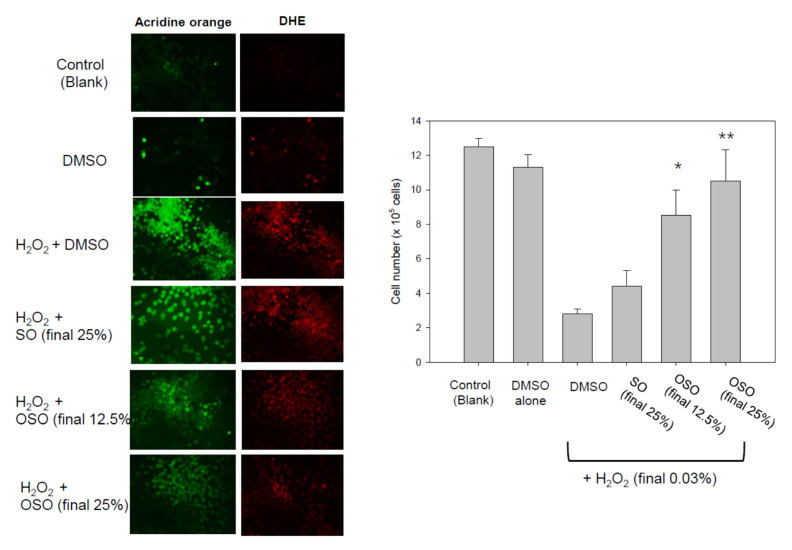
Cytoprotective effect of ozonated sunflower oil (OSO) in mouse brain microglial (BV-2) cells, showing the extent of apoptosis and reactive oxygen species (ROS) production in BV cells in the presence of H_2_O_2_ with OSO or sunflower oil (SO). Cellular apoptosis and ROS production were determined by acridine orange (Ex = 502 nm, Em = 525 nm) staining and DHE staining (Ex = 588 nm, Em = 605 nm), respectively. The graph shows the number of BV-2 cells after treatment. * *p* < 0.05 compared to SO; ** *p* < 0.01 compared to SO.

The OSO treatment facilitated the increased growth of mouse brain microglial cells via the inhibition of apoptosis, as shown in Figure 7. In the presence of H_2_O_2_ (final 0.03%), the BV-2 cells showed more significant apoptosis and ROS production, as visualized by acridine orange (AO) and DHE staining, respectively (photo of Figure 7), due to oxidative stress. The cells treated with H_2_O_2_ caused an approximately 75% decrease in cell numbers than the DMSO-treated cells, indicating severe cell death. On the other hand, the OSO-treated cells at 12.5% and 25% (final) showed a 3.0- and 3.8-times greater increase in cell numbers, respectively, than the H_2_O_2_-alone treated cells. The OSO (final 25%)-treated cells showed a 2.4 times higher number of cells than that of SO (final 25%)-treated cells, indicating that OSO facilitated more cell replication along with the suppression of apoptosis and ROS production.

AO and DHE staining showed that H_2_O_2_-treated cells caused a 5.0- and 2.9-fold higher increase in apoptosis and ROS production, respectively, than the DMSO-treated cells. In the presence of H_2_O_2_, the final 12.5% and 25% OSO treatment caused a 35% and 53% decrease in apoptosis, respectively, compared to H_2_O_2_ alone, whereas SO caused a 15% decrease in apoptosis than H_2_O_2_ alone. Concurrently, DHE staining revealed a decrease in ROS production upon OSO treatment (final 12.5% and 25%) to 33% and 43% reduction, respectively, in the presence of H_2_O_2_. Under the same dosage, the SO-treated cells showed a 2.0 times higher ROS production than the OSO-treated cells (Figure 7), suggesting that the pro-inflammatory properties of SO had become anti-inflammatory due to ozonation.

### 3.8. OSO Protected Embryo Death by H_2_O_2_

**Figure 8:**
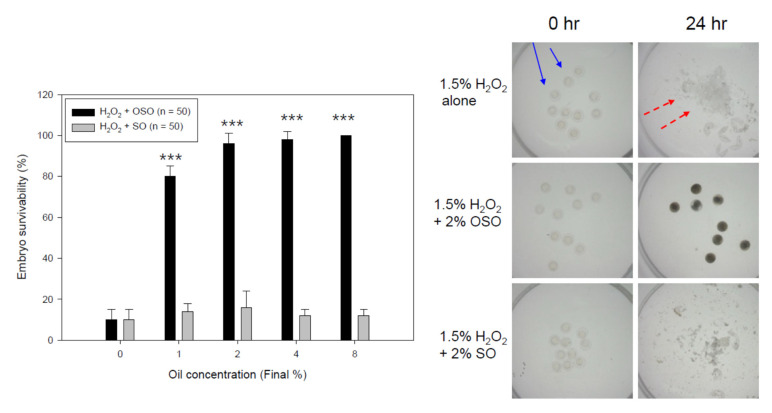
Embryo-protective effect of ozonated sunflower oil (OSO) in the presence of H_2_O_2_. *** *p* < 0.005. Blue solid arrow indicates zebrafish embryo at 0 h and red dotted arrow indicates debris of embryo at 24 h after explosion.

A H_2_O_2_ treatment (final 1.5%) resulted in the death of all embryos (4 hpf, indicated by the blue arrow) during a 24 h incubation period, with explosion and bursting (indicated by the red dashed arrow), as shown in Figure 8, indicating the severe toxicity of H_2_O_2_. In the presence of the same H_2_O_2_, a co-treatment of OSO (final 1, 2, 4, and 8%) resulted in an increase in the survivability of embryos in a dose-dependent manner, showing an increase from 80% to 100% (graph of Figure 8). On the other hand, the same SO treatment did not prevent embryo death; almost all of the embryos were killed, which was similar to the H_2_O_2_-alone treatment. The stereomicroscopic images (photos of Figure 8) also show that the SO-treated embryos disappeared along with the remaining debris after the explosion and bursting caused by H_2_O_2_. In contrast, the OSO-treated embryos showed a distinct morphology without damage. These results strongly suggest that the putative component of OSO, singlet oxygen, could neutralize the oxidative toxicity of H_2_O_2_.

### 3.9. OSO Prevented Embryo Death from Oxidative Stress by oxLDL

**Figure 9:**
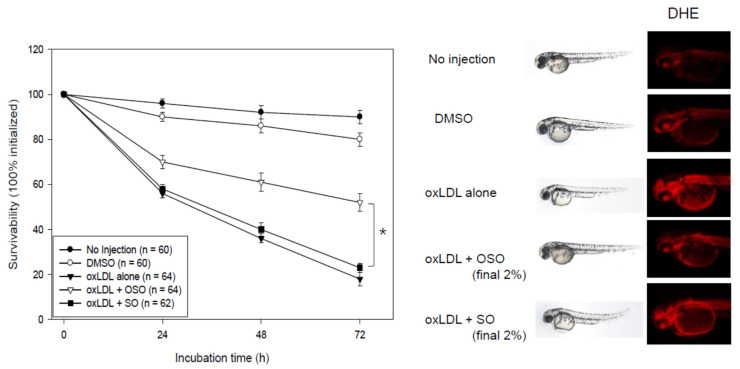
Survival of zebrafish embryos after a co-injection of oxLDL (15 ng of protein) plus ozonated sunflower oil (OSO) or sunflower oil (SO). Fluorescence image (Ex = 588 nm, Em = 605 nm) of a zebrafish embryo was obtained from dihydroethidium (DHE) staining, as described in the text. * *p* < 0.05 between OSO- and SO- injected embryo.

An injection of oxLDL-alone caused the highest death rate (up to 18% survivability) with the slowest embryo development speed during 72 h post-injection period, as shown in Figure 9. To visualize increased oxidative stress, the DHE staining showed that the oxLDL-injected embryos had the strongest red intensity, indicating an ROS production that was 1.8 times higher than that of the DMSO-injected group. In the presence of oxLDL, the co-injection of OSO (final 2%) showed 52% survivability, which was 2.8 times higher than oxLDL-alone injection. On the other hand, the SO-injection showed 23% survivability, similar to oxLDL alone. DHE staining of the SO-injected embryos showed a 1.7 times stronger red fluorescence area than the OSO-injected group. These results suggest that the OSO in the embryos exerted antioxidant activity to prevent oxLDL induced embryo death, while SO did not.

### 3.10. OSO Showed Potent Anti-Microbial Activity

**Figure 10:**
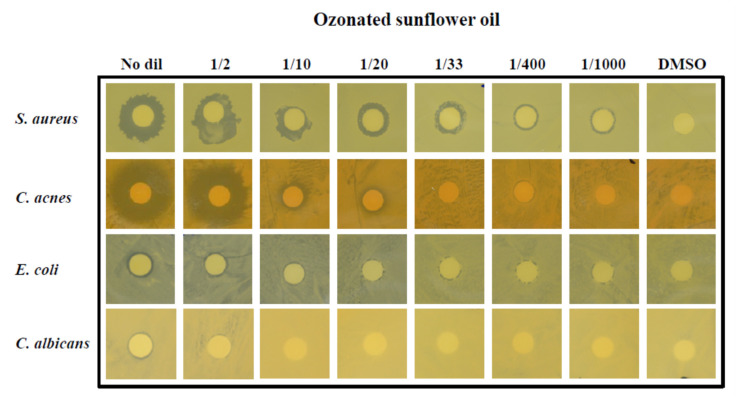
The antibacterial and antifungal effect of ozonated sunflower oil against four test strains by disk diffusion method. No dil, no dilution.

The antibacterial and antifungal effects of OSO on the four strains were evaluated using a disk diffusion assay (Figure 10). *S. aureus* had an antibacterial effect from 1/1000-dilution, the highest dilution of ozonized sunflower oil. *C. acnes* had an antibacterial effect from a 1/20 dilution of OSO.

**Figure 11:**
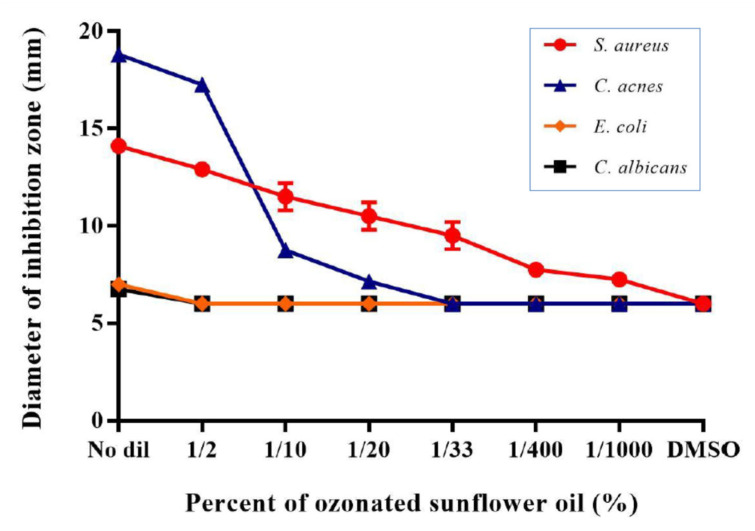
Inhibition of bacterial growth as diameter of inhibition zone in according to the concentration of ozonated sunflower oil. No dil, no dilution.

The growth of *E. coli* and *C. albicans* was inhibited without a dilution of OSO (Figure 11).

**Figure 12:**
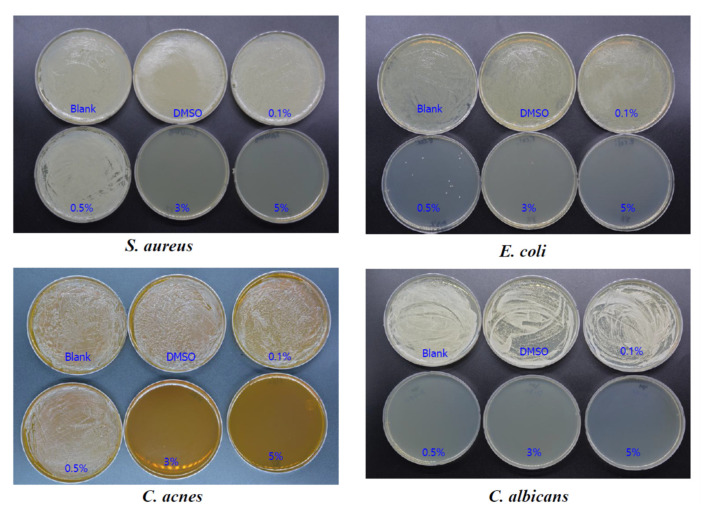
The antibacterial and antifungal effect of ozonated sunflower oil against four tested strains by minimal inhibitory concentration test.

The minimal inhibitory concentration (MIC) test showed that 0.5, 3, 0.5, and 0.1% OSO inhibited the growth of *S. aureus*, *C. acnes*, *E. coli*, and *C. albicans*, respectively, with 3, 3, 3, and 0.5% for the microbial bactericidal concentration (MBC), as shown in Figure 12 and Table 1. The antibacterial and antifungal effect of ozonated sunflower oil against the four tested strains by MIC test are also shown (C). Overall, OSO displayed potent germicidal activity for bacteria and fungi that are responsible for major skin diseases and irritable symptoms.

## 4. Discussion

Many previous studies have mainly focused on the germicidal effects and wound healing effects of OSO with potent oxidizing activity [28], even though the mechanism for wound healing is not fully understood. There is limited knowledge on what component is the source of the rejuvenation activity of OSO and how it works. Almost no explanation has been provided on how and why OSO could be used as a therapeutic agent with a wound-healing effect [29]. Furthermore, no study has evaluated the in vitro antioxidant activity of OSO and its influence on the proteins and lipids in cells and embryos. Although the basic mechanism remains to be determined, it is plausible that ozonated oil in the higher eukaryotic cell system could affect the proteins and lipids in the cell membrane via a putative reaction, such as ozonolysis. Because the serum HDL is a macromolecular complex of phospholipid, cholesterol, and protein in serum [30], it can provide a good model to study the influence of ozonated oil on protein and lipid complexes. Recently, it was suggested that ozone peroxides are pharmacologically active substances that can replace H_2_O_2_ to restore redox signaling and that can improve the antioxidant capacity [31].

OSO showed many unique abilities in vitro, such as the long-term antioxidant effects of OSO regarding radical scavenging activity and ferric ion reduction ability (Figure 1). In addition to the potent antibacterial and antifungal activity, ozone is a powerful oxidant. The current results strongly suggest that ozonated oil can be used as a potent reductant to neutralize the toxicity of ROS. The most interesting findings of this study are that an active component of OSO caused a red-shift of Trp fluorescence and loss of FI in HDL_3_ (Figure 2) via collisional quenching. OSO-treated HDL_3_ showed a more distinct band intensity and slower electromobility (Figure 3) and bigger particle size (Figure 4) than HDL_3_ alone and SO-treated HDL_3_. Furthermore, the addition of OSO into HDL_3_ caused the enhancement of HDL-associated PON-1 activity (Figure 5). These results indicate that ozonated oil treatment caused distinct structural and functional changes in HDL_3_. Putative oxygen species or singlet oxygen (^1^O_2_) in OSO could influence stronger staining ability with the Coomassie Blue because the Trp residue was more exposed to the water phase. These results work together to explain the putative enhancement of HDL structure and functionality, such as Trp movement, slower electromobility, bigger particle size, and higher PON-1 activity.

The OSO treatment facilitated more growth of murine macrophage cells (Figure 6) and prevented the cell death of mouse brain microglia from H_2_O_2_ toxicity (Figure 7). In the zebrafish embryo model, the co-presence of OSO prevented embryo death from H_2_O_2_ toxicity (Figure 8) and the toxicity of oxLDL (Figure 8). The low dosage of OSO (<3%) inhibited the growth of three kinds of pathogenic bacteria (*S. aureus*, *C. acnes*, *E. coli*) and one type of fungus (*C. albicans*), as shown in Table 1 and Figure 10.

Previous studies have suggested that OSO reduced oxidative damage in a rat model as well as ethanol-induced ulcers [32] and indomethacin-induced gastric mucosa [33], even though the mechanism was not elucidated. In the gastro mucosa of the ethanol-induced ulcer model, OSO-treated mice showed 1.24- and 1.18-times higher glutathione peroxidase and superoxide dismutase levels, respectively, than the SO-treated mice. These reports showed good agreement with the current results that OSO exerted radical scavenging and ferric ion reduction ability with enhanced PON-1 activity. The current study strongly suggests that a putative active ingredient of OSO could exert wound-healing and antioxidant activity. The more oxidized lipoproteins showed faster electromobility in agarose gel. OSO-treated HDL_3_ showed much slower electromobility with a stronger band intensity than SO-treated HDL_3_. These results suggested that the OSO treatment caused an increase in the HDL structure and functionality regarding the electronic status and protein stability via a putative mechanism.

It is already known that small molecules, such as oxygen, iodide, and acrylamide, can cause the collisional quenching of Trp, which depopulates the excited state, leading to decreased fluorescence lifetimes. Excited-state oxygen (^1^O_2_) quenches the fluorescence of an organic chomophore at the diffusion-controlled limit [34]. Because OSO contains ozone-derived oxygen species, in the same context, the OSO treatment caused a remarkable and instant decrease in fluorescence intensity, even though the buried Trp had moved and was exposed to the aqueous phase, known as the red-shift in WMF. To the best of the authors’ knowledge, this is the first study to report that ozonated oil could induce the collisional quenching of Trp in apoA-I and HDL_3_ via a putative interaction because singlet oxygen (^1^Δ_g_) can react with the amino acid residues Tyr, His, and Trp, which have high electron density. Singlet oxygen is also very susceptible to removal by Trp, resulting in the bleaching of fluorescence, a decrease in the intensity of Trp fluorescence [35].

The singlet oxygen in OSO is highly hydrophobic, and Trp108 in HDL is also located in a hydrophobic local environment. A reaction between OSO and Trp108 must occur though a complex formed by a diffusion-dependent encounter of the two reactants. For Trp, the addition of ^1^O_2_ produced short-lived endoperoxides that can ring open to produce hydroperoxides at ring positions. The Trp fluorescence in HDL_3_ and apoA-I strongly depends on the movement of Trp108, which was buried in the hydrophobic core of the proteins [36]. The greater red-shift of WMF means more exposure of Trp108 to the hydrophilic phase upon binding with OSO. This suggests that a singlet oxygen or putative oxygen species in OSO could help induce a change in the three-dimensional structure, especially in the hinge-mobile region of HDL_3_. A “hinge” or “mobile region” between residues 100 and 183 of the apoA-I lipid-bound state was implicated in structural rearrangements and functional changes in HDL [37]. The amphipathic helices of apoA-I are believed to participate in the structural rearrangements of HDL when their lipid and apolipoprotein contents change during metabolism [38]. Therefore, the fluorospectroscopic properties, paraoxonase ability, and particle size in HDL change after treatment with either OSO or SO. In the same context, the putative oxygen species in OSO induced remarkable collisional quenching (Figure 2B) of the intrinsic fluorescence of Trp as in the other small molecules, such as oxygen, iodide, and acrylamide.

Interestingly, OSO-treated HDL_3_ showed a higher PON-1 activity with bigger particle size than those of HDL_3_ alone or SO-treated HDL_3_. Although the precise mechanism remains to be investigated, the current results might explain the effects of ozone therapy that have been reported elsewhere [39], including the up-regulation of antioxidants enzymes, the activation of immune systems, and stimulation of the neuroendocrine system. These results also are also in good agreement with previous report [40] where a healthy individual with higher HDL-C and bigger HDL particles showed higher PON-1 activity that took place in a size-dependent manner. More interestingly, the higher PON-1 was also associated with the lower C-reactive proteins (CRP) and the smaller carotid intima media thickness [40].

In contrast to the germicidal activity, less attention has been paid to this protective effect of OSO on cells and embryos against oxidative and inflammatory stress. These unique features of OSO in the HDL structure and protection for cells and embryos can be applied to develop an evaluation method to compare the quality of ozonated oil. There has been no method to compare the quality and stability from various ozonated oils, such as mixed with sunflower oil, olive oil, flaxseed oil, and coconut oil. The current characterization methods of the ozonated oils are limited to physicochemical determination, such as the total unsaturation extent, iodine value, peroxide value, acidity value, and viscosity. Therefore, the biochemical characterization method from this study could be a useful tool to evaluate and compare the functionality and quality of many commercial ozonated oils.

Further studies will be needed to investigate the precise mechanism to determine why OSO can enhance the HDL-associated PON activity (Figure 5). An increase in PON activity should be accompanied by protecting cells and tissues because the PON activity has potent cholesterol efflux antioxidant and stimulant activity [41]. Recently, the PON-1 activity in healthy HDL has been indicated to be critical to the antiviral activity of HDL that is needed to kill SARS-CoV-2 [23]. Moreover, the antiviral activity of HDL is strongly dependent on the apoA-I content and apolipoprotein compositions [42]. Similarly, ozone therapy significantly improved PON-1 activity in HDL from coronary artery disease patients who were experiencing a decrease in lipid-peroxidation and LDL-oxidation [43]. These results showed good agreement with previous human trials [43,44] and animal experiments [4,45] using ozone therapies, such as the elevation of HDL-C and PON-1 activity, even though the detailed mechanism needs to be investigated.

## 5. Conclusions

OSO exerted potent antioxidant activity in vitro and facilitated cell growth with the inhibition of ROS production. The co-treatment of OSO prevented embryo death from exogenous oxidative stress, H_2_O_2_, and oxLDL. OSO could change the Trp fluorescence of HDL and could enhance PON activity. The results showed that OSO has antimicrobial activity, antioxidant activity, and protective effects on animal cells and embryos. These results can be applied to develop evaluation methods to compare the antioxidant ability and wound-healing activity of ozonated oil.

## Figures and Tables

**Table 1:** Antimicrobial activity of OSO.

Strains	ATCC No	MIC (%)	MBC (%)
*Staphylococcus aureus*	ATCC 6538	0.5	3
*Cutibacterium acnes*	ATCC 6919	3	3
*Escherichia coli* BL21	ATCC 25922	0.5	3
*Candidia albicans*	ATCC 90028	0.1	0.5

MIC, minimum inhibitory concentration; MBC, microbial bactericidal concentration.

## Data Availability

Data is contained within the article.
